# Complete Genome Sequence of *Nitrospina watsonii* 347, Isolated from the Black Sea

**DOI:** 10.1128/mra.00078-23

**Published:** 2023-03-21

**Authors:** Linnea F. M. Kop, Hanna Koch, Eva Spieck, Theo van Alen, Geert Cremers, Holger Daims, Sebastian Lücker

**Affiliations:** a Department of Microbiology, Radboud Institute for Biological and Environmental Sciences, Radboud University, Nijmegen, The Netherlands; b University of Vienna, Centre for Microbiology and Environmental Systems Science, Department for Microbiology and Ecosystem Science, Division of Microbial Ecology, Vienna, Austria; c University of Vienna, Doctoral School in Microbiology and Environmental Science, Vienna, Austria; d Department of Microbiology and Biotechnology, University of Hamburg, Hamburg, Germany; Montana State University

## Abstract

Here, we present the complete genome sequence of Nitrospina watsonii 347, a nitrite-oxidizing bacterium isolated from the Black Sea at a depth of 100 m. The genome has a length of 3,011,914 bp with 2,895 predicted coding sequences. Its predicted metabolism is similar to that of Nitrospina gracilis with differences in defense against reactive oxygen species.

## ANNOUNCEMENT

Members of the family *Nitrospinaceae* within the phylum *Nitrospinota* constitute the prevalent nitrite oxidizers in the marine environment (1, 2). Here, we report the complete genome sequence of Nitrospina watsonii 347, which was isolated from an enrichment culture started in 1969 with a Black Sea water sample from a depth of 100 m and was described in 2014 (3).

*N. watsonii* was obtained as an active culture from the collection of nitrite-oxidizing bacteria at Hamburg University and grown in the dark at 28°C in a synthetic seawater medium (https://doi.org/10.17504/protocols.io.81wgby1zovpk/v1). Next, 3 mM nitrite was added to the culture and replenished whenever it was consumed completely.

DNA was isolated from the culture using the DNeasy blood and tissue kit (Qiagen). For short-read sequencing, libraries were prepared using the Nextera XT kit (Illumina), quality checked using the Agilent 2100 bioanalyzer with the high-sensitivity (HS) DNA kit, and quantified using a Qubit instrument with the double-stranded DNA (dsDNA) HS assay kit (Thermo Fisher Scientific Inc.). Paired-end sequencing of 2 × 300 bp was performed using the Illumina MiSeq sequencer with MiSeq reagent kit v3, producing 11,829,056 sequence pairs with an *N*_50_ value of 300 bp. For Nanopore library preparation, the ligation sequencing kit 1D (SQK-LSK108) and the native barcoding expansion kit (EXP-NBD104) were used (Oxford Nanopore Technologies). The libraries were loaded on a flow cell (R9.4.1) and run on a MinION device (Oxford Nanopore Technologies). Basecalling was done using albacore v2.1.10 with the basecalling model template_r9.4_450bps_5mer_raw.jsn (Oxford Nanopore Technologies). In total, 290,544 sequence reads were obtained with an *N*_50_ value of 8,228 bp. Manufacturer instructions were followed for all protocols.

Nanopore reads were assembled using canu v.1.8 (4) (options: genomeSize = 3.6m, stopOnReadQuality=false, stopOnLowCoverage = 5) and were subsequently mapped against the assembly using minimap2 with default settings (5) to polish the canu assembly with racon v1.3.1 (6). Illumina reads were trimmed and filtered using BBDuk from BBTools (DOE Joint Genome Institute, Walnut Creek, CA, USA; parameters used: k = 23 mink = 11 hdist = 1 ktrim=r tbo qtrim=rl trimq = 17 maq = 20 maxns = 0 minlen = 150 tossjunk=t). A hybrid assembly was then constructed with unicycler v0.4.4 (7), using the existing long-read assembly from canu. The resulting circular genome had a mean genome coverage of 54.32x and was centered around the origin of replication using the “start_gene” option with the *dnaA* gene of Nitrospina gracilis (CCQ89934.1) (8) and a minimum required BLAST identity of 60%.

The circular genome of *Nitrospina watsonii* 347 has a total length of 3,011,914 bp, with 2,895 predicted coding sequences based on the MicroScope annotation platform (9) and an average GC content of 57.16%. The genome contains 1 complete rRNA operon and in total 45 tRNA genes (1 to 5 for each of the 20 amino acids). The core metabolism is as described for Nitrospina gracilis 3/211 (8), but the organisms differ in their potential for defense against reactive oxygen species, as *N. watsonii* carries a superoxide reductase not identified in *N. gracilis.*

### Data availability.

The annotated genome of *Nitrospina watsonii* 347 has been deposited at ENA under accession number ERZ13641452 (PRJEB56043). The version described in this paper is the first version. The accessions for the raw reads are ERR10676989 (long reads; Oxford Nanopore) and ERR10676991 (short reads; Illumina MiSeq).
